# Long-term efficacy and safety of combined prolonged-release oxycodone and naloxone in the management of non-cancer chronic pain

**DOI:** 10.1111/j.1742-1241.2010.02360.x

**Published:** 2010-05

**Authors:** A Sandner-Kiesling, P Leyendecker, M Hopp, L Tarau, J Lejcko, W Meissner, P Sevcik, M Hakl, R Hrib, R Uhl, H Dürr, K Reimer

**Affiliations:** 1 Department of Anaesthesiology and Intensive Care Medicine, Medical UniversityGraz, Austria; 2 Mundipharma Research GmbH & Co. KG, HoehenstrasseLimburg (Lahn), Germany; 3 Facharztzentrum MedicumLangenbeckplatz, Wiesbaden, Germany; 4 Centre for Pain Treatment, Department of Anaesthesiology and Intensive Care, University Hospital PilsenAlej Svobody, Pilsen-Lochotin, Czech Republic; 5 Clinic of Anaesthesiology and Intensive Care, Friedrich-Schiller-UniversityJena, Germany; 6 Department of Anaesthesiology and Intensive Care, Faculty of Medicine, Masaryk University Brno and Teaching Hospital of St AnnaPekarska, Brno, Czech Republic; 7 Medical Faculty, University of Witten/HerdeckeWitten, Germany

## Abstract

**Objective:**

The aim of this study was to assess safety and efficacy of fixed combination oxycodone prolonged release (PR)/naloxone PR in terms of both analgesia and improving opioid-induced bowel dysfunction (OIBD) and associated symptoms, such as opioid-induced constipation (OIC), in adults with chronic non-cancer pain.

**Study design:**

These were open-label extension studies in which patients who had previously completed a 12-week, double-blind study received oxycodone PR/naloxone PR for up to 52 weeks. The analgesia study assessed pain using the modified Brief Pain Inventory-Short Form (BPI-SF). The bowel function study assessed improvements in constipation using the Bowel Function Index (BFI).

**Results:**

At open-label baseline in the analgesia study (*n* = 379), mean score [± standard deviation (SD)] for the BPI-SF item ‘average pain over the last 24 h’ was 3.9 ± 1.52, and this remained low at 6 months (3.7 ± 1.59) and 12 months (3.8 ± 1.72). Mean scores for BPI-SF item ‘sleep interference’, and the BPI-SF ‘pain’ and ‘interference with activities’ subscales also remained low throughout the 52-week study. In the bowel function study (*n* = 258), mean BFI score (± SD) decreased from 35.6 ± 27.74 at the start of the extension study to 20.6 ± 24.01 after 12 months of treatment with oxycodone PR/naloxone PR. Pain scores also remained low and stable during this study. Adverse events in both extension phases were consistent with those associated with opioid therapy; no additional safety concerns were observed.

**Conclusion:**

Results from these two open-label extension studies demonstrate the long-term efficacy and tolerability of fixed combination oxycodone PR/naloxone PR in the treatment of chronic pain. Patients experienced clinically relevant improvements in OIBD while receiving effective analgesic therapy.

**What's known**

The fixed combination of oxycodone PR/ naloxone PR was shown to be tolerable and effective in providing analgesia and improving bowel function in a 12 weeek randomised, controlled study setting.

**What's new**

The fixed combination of oxycodone PR and naloxone PR is a safe and efficacious agent also for the long-term treatment of chronic pain.

## Introduction

Almost one in five adults within the European Union suffers from chronic pain (1), imposing a significant burden on their quality of life (QoL). Persistent chronic pain is associated with depression and anxiety, interference with work and personal relationships and loss of independence (2).

As recommended by the World Health Organization, opioids have become the established treatment for moderate-to-severe chronic cancer pain (3) and, in recent years, have also become a mainstay for the treatment of chronic non-cancer pain. Oxycodone is a semi-synthetic, opioid analgesic that has demonstrated effectiveness in treating cancer and non-cancer related pain (4–9). However, the primary disadvantage associated with these agents is the development of opioid-induced bowel dysfunction (OIBD) in many patients, which commonly manifests as significant constipation (10).

Opioid-induced bowel dysfunction is a consequence of the action of opioids on receptors within the gastrointestinal (GI) tract, which reduce GI motility, inhibit secretion, increase absorption, affect blood flow and increase anal sphincter tone (10). As a result, patients can experience a range of symptoms such as straining, incomplete evacuation, bloating, abdominal distension and increased gastric reflux (10). Constipation is the most frequently-reported adverse event in patients receiving opioid treatment (11). Unlike most adverse events associated with opioid use, which subside with chronic use, opioid-induced constipation (OIC) persists in many patients (12). The pain and discomfort caused by OIC can cause patients to reduce or even discontinue their opioid therapy (13), resulting in inadequate analgesia and further impairment of QoL (14,15).

Current management strategies for OIC are non-specific and often ineffective (16). Laxatives can improve symptoms in some patients; however, as this strategy fails to address the underlying opioid receptor-mediated mechanism of bowel dysfunction that leads to constipation in these patients, a substantial number do not achieve adequate relief of symptoms (10,13). In addition, laxatives can be associated with several drawbacks (10,17), and furthermore, long-term laxative use can be associated with damage to the muscular function of the bowel; nutritional deficits in terms of loss of water, vitamins and minerals; and kidney stones or renal failure, in addition to modifying the effects of other medicines.

Prevention of OIC, and bowel dysfunction in general, is considered to be a more effective therapeutic strategy than merely treating the symptoms as they occur (13). One approach to targeting the underlying cause of OIC is the oral co-administration of opioids and opioid antagonists with limited systemic bioavailability. By acting locally within the gut to block opioid action, the opioid antagonist would prevent or minimise OIBD, while the lack of systemic activity would mean no reduction in the central analgesic effects of the opioid.

Naloxone is an opioid-receptor antagonist that, when administered orally, has a very low systemic bioavailability of < 3% (16) because of extensive first-pass hepatic metabolism (18). As a result, naloxone acts almost exclusively on opioid receptors in the GI tract (19). Results from a pharmacokinetic study in healthy subjects demonstrated that co-administration of oxycodone prolonged release (PR)/naloxone PR in a fixed dose combination does not significantly affect the bioavailability of either of its constituents (20). As such, the oral co-administration of oxycodone PR/naloxone PR has been shown to provide an effective analgesia for patients with severe chronic pain, and to significantly reduce the impact of OIC (21).

The efficacy and tolerability of the fixed combination of oxycodone PR/naloxone PR has been shown in two Phase III, double-blind, randomised controlled clinical trials, one focusing on analgesic efficacy and the other focusing on bowel function. The first showed oxycodone PR/naloxone PR to be superior to placebo in terms of analgesic efficacy while also providing significant benefits in terms of bowel function (22). Furthermore, the addition of naloxone PR to oxycodone PR in a fixed combination formulation did not negatively impact on the analgesic efficacy of oxycodone PR. In the second Phase III trial, patients receiving the fixed combination of oxycodone PR/naloxone PR experienced significant improvements in OIC compared with those receiving oxycodone PR, with comparable analgesia (23). In addition, clinically relevant improvements in bowel function have been observed in patients with chronic pain treated with oxycodone PR/naloxone PR, compared with oxycodone PR alone (24). Naloxone PR/oxycodone PR has also been shown to improve patient assessment of analgesic opioid therapy for severe chronic pain, in terms of both efficacy and tolerability (25).

This report presents the results of the open-label extensions of the aforementioned two Phase III studies (22,23), one examining the long-term analgesic efficacy of oxycodone PR/naloxone and the other investigating bowel function. The tolerability of the fixed combination of oxycodone PR/naloxone PR in the treatment of patients with moderate-to-severe non-cancer pain was also assessed in both open-label extension phases.

## Methods

These were uncontrolled, open-label, extension phase studies in patients with non-cancer pain, who had completed one of two previous randomised, controlled, 12-week studies (22,23), performed to assess the efficacy and safety of oxycodone PR/naloxone PR for an additional 52 weeks. Both studies were conducted in accordance with the Declaration of Helsinki (26) and all of its accepted amendments to date and all relevant German laws, as well as complying with the International Conference for Harmonisation Guideline for Good Clinical Practice (27) and the European Union Clinical Trials Directive 2001/20/EC (28). Written consent was obtained from all participants prior to study commencement.

### Patient population

All patients who completed one of the previous double-blind studies (22,23), who required daily opioid therapy and who were likely to benefit from treatment for the duration of the study were eligible to enter the respective extension phase. These patients were males and females who were at least 18 years old with a history of moderate-to-severe, non-malignant pain that had been effectively managed with daily opioid therapy for at least 2 weeks before entry into the double-blind phase of one of the studies. Patients with a history of hypersensitivity to oxycodone, naloxone or any related product, a diagnosis of cancer (not including basal cell carcinoma), clinically significant cardiovascular, renal, hepatic, GI or psychiatric disease that would have placed the patient at risk upon exposure to the study medication or that could confound the analysis and/or interpretation of the study results were excluded from the study. Other exclusion criteria included active alcohol or substance abuse, and abnormal liver function tests. In the analgesia study, patients with a history of more than two lower back surgeries and those receiving the equivalent of < 10 mg or > 40 mg oxycodone per day were also excluded.

At the start of the double-blind phase of the bowel function study, patients had to be experiencing constipation caused or aggravated by opioids, be willing to discontinue their current laxative regimen and comply with the use of oral bisacodyl as a laxative rescue medication. Laxative rescue medication was permitted no sooner than 72 h after the patient’s most recent bowel movement; however, if patients experienced discomfort during this period, they could take bisacodyl 5 mg as a laxative earlier than 72 h after their most recent bowel movement, as required, to treat constipation. The maximum total amount of oral bisacodyl permitted was five doses within seven consecutive days. Patients taking daily fibre supplementation or bulking agents were eligible for inclusion if they could be maintained on a stable dose and regimen throughout the study and were considered able to maintain adequate hydration.

### Study design

**Figure 1:**
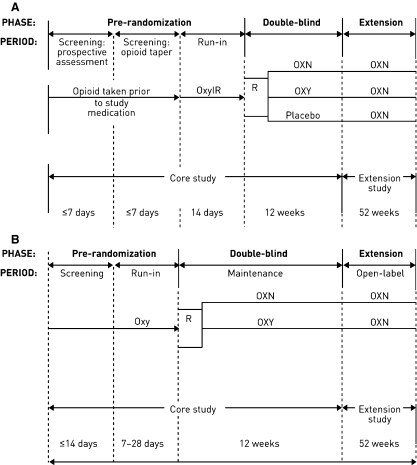
Study design of (A) the analgesia study and (B) the bowel function study. OXN, oxycodone PR/naloxone PR; OXY, oxycodone PR; OXYIR, oxycodone immediate-release; R, randomisation

In the extension phase of both studies, patients’ pain was treated with open-label study medication (oxycodone PR/naloxone PR) for up to 12 months (Figure 1). In the analgesia extension study, all patients were initially switched to 20/10 mg oxycodone PR/naloxone PR per day to prevent patients who had received placebo in the double-blind study from being given an initial high dose of opioid. In the bowel function study, the initial starting dose of oxycodone PR/naloxone PR was the effective analgesic dose, based on the oxycodone dose that the patient was receiving at the end of the double-blind phase. Dose titration up to 80/40 mg oxycodone PR/naloxone PR per day was permitted in both studies at the discretion of the investigator. In both studies, immediate-release (IR) oxycodone was provided as study medication during the first 7 days of the extension phase for titration. After this period investigators could prescribe also other analgesic rescue medicine, if needed. In the bowel function study, bisacodyl was also provided as rescue medication only during the first 7 days of the extension phase. Any additional laxative treatment was by consultation with the investigator.

### Efficacy outcomes and assessments

The extension studies were conducted primarily to assess the long-term safety of the fixed combination of oxycodone PR/naloxone PR in the treatment of chronic non-cancer pain. No defined primary end-point was set; however, analgesic efficacy and bowel function were assessed.

#### Analgesia study

The objective of the analgesia study was to assess pain and interference of pain with activities during treatment with oxycodone PR/naloxone PR, based on the modified Brief Pain Inventory–Short Form (BPI-SF) (29). The modified BPI-SF consists of 12 questions designed to assess the severity of patients’ pain and the impact of pain on daily functions. The pain subscale consists of the first four questions [numerical rating scale (NRS) 0–10: 0 = no pain; 10 = pain as bad as you can imagine]. The interference subscale consists of questions 6–12 (NRS 0–10: 0 = does not interfere; 10 = completely interferes) and question 5 reflects pain relief (NRS 0–10: 0 = no relief; 10 = complete relief). The study also assessed changes in the dose of oxycodone PR/naloxone PR during the study period. Study medication intake (time and dose) was recorded by the investigators; change in dose from the randomised dose to the end of the extension phase, as well as after 2 weeks of the extension phase, was assessed as an efficacy parameter.

#### Bowel function study

The objective of this study was to assess whether patients with moderate-to-severe non-cancer pain taking oxycodone PR/naloxone PR had improvements in bowel function, as measured by the validated Bowel Function Index (BFI) (30, 32) at each study visit over 52 weeks. The BFI score of each patient was defined as the mean score of three distinct components: ease of defaecation [numerical analogue scale (NAS) 0–100: 0 = easy/no difficulty; 100 = severe difficulty]; feeling of incomplete bowel evacuation (NAS 0–100: 0 = not at all, 100 = very strong); and judgement of constipation (NAS 0–100: 0 = not at all; 100 = very strong). Each question referred to the patient’s experience during the past 7 days, with higher scores indicating poor bowel function. Average pain over the last 24 h was also assessed at each study visit using the Pain Intensity Scale (NRS 0–10), and frequency of rescue medication and laxative use was measured.

### Safety assessments

Safety assessments consisted of monitoring all adverse events (AEs), including serious adverse events (SAEs); monitoring haematology, blood chemistry and urine values; periodic measurement of vital signs and electrocardiograms (ECGs); and physical examinations. In the bowel function study, symptoms of opioid withdrawal were also assessed using the Subjective Opiate Withdrawal Scale (SOWS) (31).

### Statistical analysis

The extension study population consisted of all patients who received at least one dose of oxycodone PR/naloxone PR in the extension phase. In the analgesia study, a subpopulation was defined *post hoc*, consisting of patients who received oxycodone PR/naloxone PR > 40 mg/20 mg per day on > 7 days consecutively. In the bowel function study, a *post hoc* analysis was conducted to examine changes in BFI score according to treatment received in the double-blind phase.

#### Analgesia study efficacy analysis

Summary statistics were provided for each single item and each subscale of the BPI-SF using the last observation carried forward (LOCF) method for missing values. Summary statistics of average pain over 24 h, the pain subscale, the sleep interference and the interference subscale were also displayed for observed values without any missing values. Changes in dose were assessed using a shift table with numbers of changes from the dose at the start of the double-blind phase to the oxycodone PR/naloxone PR dose at the end of the extension phase, as well as the oxycodone PR/naloxone PR dose after 2 weeks of the extension phase. Changes in dose after 2 weeks of the extension phase were grouped according to double-blind phase treatment.

#### Bowel function study efficacy analysis

Continuous efficacy variables were summarised by *n* (i.e. the number of non-missing values), mean (i.e. arithmetic average) and standard deviation (SD), whereas number and percentage were used to summarise categorical efficacy variables.

#### Safety analysis

For the safety data analyses, all continuous variables were summarised by *n* (i.e. the number of non-missing values), mean (i.e. arithmetic average) and SD. The number and percentage of observed levels were reported for all categorical measures. AEs were classified by system organ class and Medical Dictionary for Regulatory Activities (MedDRA)-preferred term. The incidence of AEs considered as having a causal relationship to study medication (unlikely, possible, probable or definite) was also recorded. In the analgesia study, the incidence of GI AEs and nervous system disorders was recorded for 0–3 months, 3–6 months, 6–9 months, 9–12 months, > 12 months and follow up.

## Results

### Analgesia study

**Figure 2:**
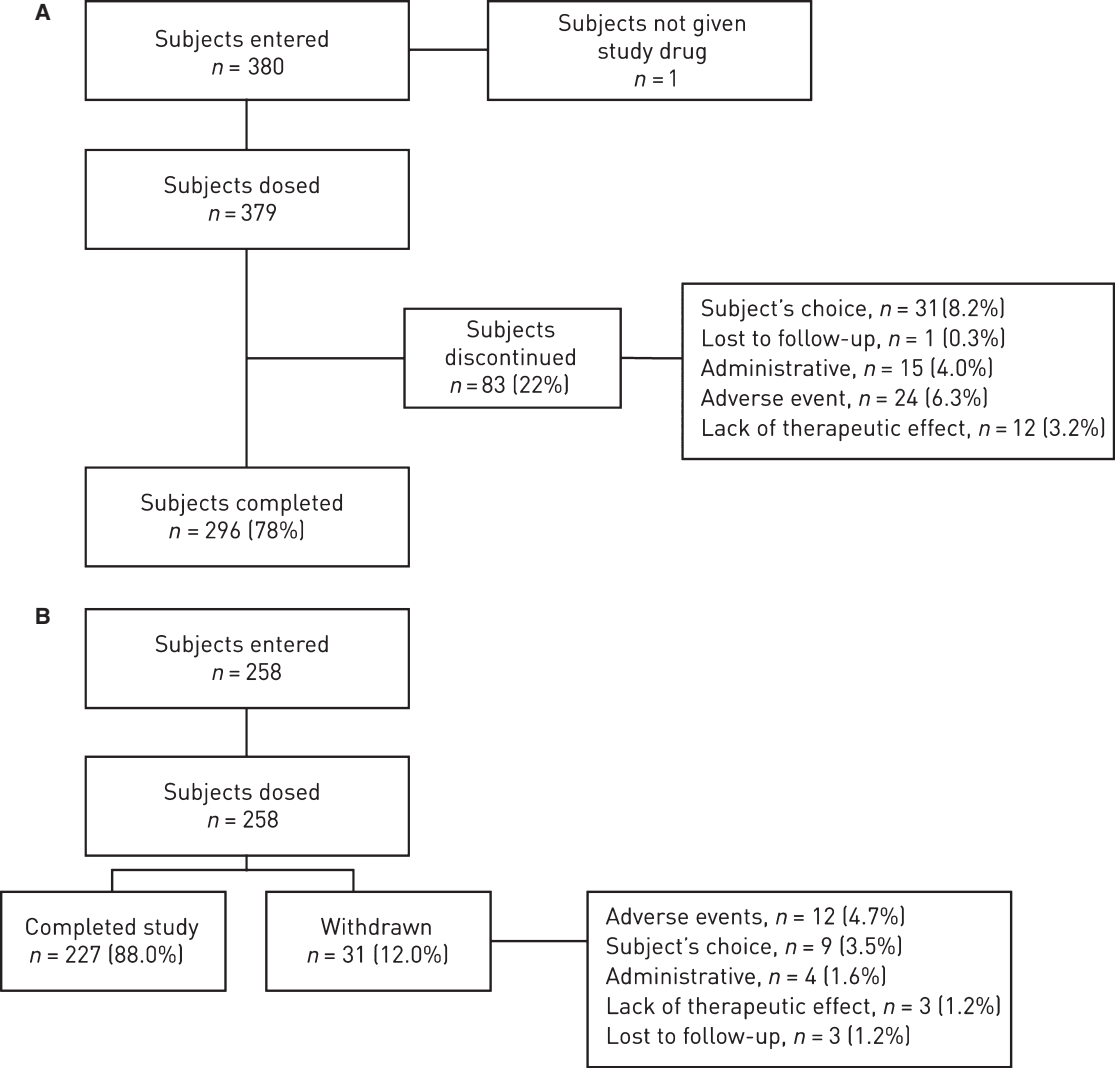
Patient disposition in (A) the analgesia study and (B) the bowel function study

Of the 464 patients who were initially randomised, 463 received study medication and entered the double-blind phase. Of these patients, 380 (81.9%) continued to the extension phase and 379 received study medication. The majority of patients (78%) completed the 12-month study, with 86% remaining in the study at 6 months. Only 83 patients (22%) had discontinued the study by 12 months; the majority of these discontinuations were resulting from the patients’ choice (31 patients, 8.2%), administrative error (15 patients, 4.0%) or patients being lost to follow up (one patient, 0.3%) (Figure 2A). AEs were responsible for 24 (6.3%) patient discontinuations, and 12 (3.2%) discontinuations were resulting from lack of therapeutic effect.

Patients were 56.2 years in average, with 39% male and 61% female; all were Caucasian (Table 1). The average equivalent dose of oxycodone taken in the full analysis population during the extension phase was 40.9 mg/day, and the mean exposure to study medication was 320.5 days (median = 365 days). In the subpopulation of patients who received oxycodone PR/naloxone PR > 40 mg/20 mg per day on > 7 days consecutively, the average dose of oxycodone was 67.8 mg/day, and the mean number of days patients were on doses > 40 mg/20 mg oxycodone PR/naloxone PR was 244.5 days.

**Table 1:** Patient baseline demographics

	Analgesia study (*n* = 379)	Bowel function study (*n* = 258)
**Sex, *n* (%)**		
Male	148 (39)	102 (39.5)
Female	231 (61)	156 (60.5)
**Age, years**		
Mean ± SD	56.2 ± 10.88	58.4 ± 11.91
**Weight, kg**		
Mean ± SD	83.2 ± 17.87	85.4 ± 18.56

SD, standard deviation.

#### Efficacy evaluation

**Figure 3:**
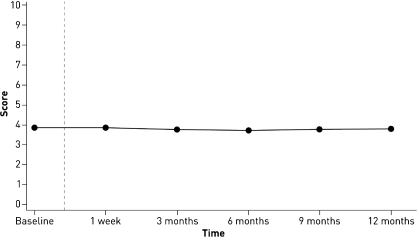
Brief Pain Inventory-Short Form item ‘average pain over the last 24 h’– mean score by visit: analgesia study (*n* = 379). BPI-SF, Brief Pain Inventory – Short Form

The objective of the extension study was to assess the long-term analgesic efficacy of oxycodone PR/naloxone PR for up to 12 months. After entering the extension phase, the mean pain score (± SD) for the BPI-SF item ‘average pain over the last 24 h’ was 3.9 ± 1.52 (Week 1), which was comparable with the mean score at the end of the double-blind phase (Table 2). Mean pain scores (±SD) remained low and stable over 6 months (3.7 ± 1.59) and 12 months (3.8 ± 1.72), indicating effective long-term analgesia with oxycodone PR/naloxone PR (Figure 3). At Week 1, the mean score on the BPI-SF pain subscale (±SD) was 15.3 ± 6.18, and this remained low and stable over 6 months (14.6 ± 6.67) and 12 months (14.8 ± 6.93), confirming effective long-term analgesia with oxycodone PR/naloxone PR (Table 2).

**Table 2:** Mean change in BPI-SF items by study visit [analgesia study; LOCF; extension population (*N* = 379)]

	BPI-SF item			
Visit	Average pain over the last 24 h (0–10) Mean ± SD	Sleep quality item (0–10) Mean ± SD	Pain subscale (0–40) Mean ± SD	Interference subscale (0–70) Mean ± SD
End of double-blind study	3.8 ± 1.48	3.1 ± 2.67	15.3 ± 6.09	21.6 ± 13.10
Week 1	3.9 ± 1.52	2.9 ± 2.52	15.3 ± 6.18	21.2 ± 12.54
3 months	3.8 ± 1.60	3.0 ± 2.48	14.7 ± 6.55	22.2 ± 12.80
6 months	3.7 ± 1.59	3.2 ± 2.50	14.6 ± 6.67	22.4 ± 12.77
9 months	3.7 ± 1.66	3.3 ± 2.64	14.8 ± 6.70	23.0 ± 13.23
12 months	3.8 ± 1.72	3.1 ± 2.48	14.8 ± 6.93	23.0 ± 13.00

BPI-SF, Brief Pain Inventory – Short Form; LOCF, last observation carried forward; SD, standard deviation.

The mean score (±SD) for the BPI-SF item ‘sleep interference’ was 2.9 ± 2.52 after switching to the extension phase (Week 1), which was comparable with the mean score at the end of the double-blind phase (Table 2). Mean sleep interference scores (±SD) remained low and stable over 6 months (3.2 ± 2.50) and 12 months (3.1 ± 2.48), which correlated well with the low BPI-SF pain scores, indicating a beneficial effect of oxycodone PR/naloxone PR on sleep quality. After entering the extension phase (Week 1), the mean BPI-SF interference subscore (±SD) was 21.2 ± 12.54, which remained low and stable over 6 months (22.4 ± 12.77) and 12 months (23.0 ± 13.00); this correlated well with the low BPI-SF pain scores and indicated a positive effect on pain with activities.

In the subgroup of patients using daily oxycodone PR/naloxone PR doses greater than 40/20 mg, the mean average pain score (±SD) at the end of the double-blind phase was 4.2 ± 1.4; this remained stable throughout the study (varying between 4.1 and 4.3), being 4.2 ± 1.74 at 12 months. These pain scores were comparable with those of the total extension population, and no clinically relevant differences could be observed throughout the 12-month extension phase.

After the first 2 weeks of the extension phase, the majority of patients remained on a dose of oxycodone PR/naloxone PR that was comparable with the dose they had received in the double-blind phase. A total of 53.8% of patients remained on the same dose as they received in the double-blind phase. The percentage of patients who had a decrease or increase in dose was comparable between, and independent of, the different double-blind phase treatment groups from which the patients had been switched (Table 3). The mean total daily dose of oxycodone (±SD) increased slightly from 35.6 ± 16.53 mg after 2 weeks to 43.7 ± 22.53 mg at the end of the extension phase, indicating a natural progression of the underlying chronic pain condition over this period.

**Table 3:** Number of patients with a dose increase or decrease after the first 2 weeks of the extension phase grouped by double-blind medication: analgesia study

Treatment during double-blind phase	Decrease in dose, *n* (%)	Increase in dose, *n* (%)
**Oxycodone PR/naloxone PR**		
20 mg/10 mg	0 (0)	22 (37)
40 mg/20 mg	17 (24)	19 (27)
**Oxycodone PR**		
20 mg	3 (5)	24 (37)
40 mg	8 (15)	18 (33)
**Placebo**		
20 mg	2 (3)	33 (46)
40 mg	9 (17)	20 (38)

PR, prolonged release.

#### Safety evaluation

Overall, the incidence of AEs in the extension phase (68%) was comparable with that in the double-blind phase (oxycodone PR/naloxone PR 55.8%; oxycodone PR 53.0%; placebo 52.5%), taking into account the longer observation period. Most AEs were mild or moderate, and the incidence of severe AEs was low (13%). For the majority of AEs (62%), no action was taken regarding the study drug. In the subgroup of patients using oxycodone PR/naloxone PR doses greater than 40/20 mg daily, the incidence of AEs was slightly higher than in the overall population, during the extension phase (71.6 vs. 68.0% respectively).

The incidence of AEs considered as having a causal relationship to study medication was 38% and the number of AEs leading to treatment discontinuation was 6.3%. Constipation (9.2%), nausea (7.7%), back pain (6.3%) and depression (6.3%) were the most frequently reported AEs (Table 4). The incidence of GI AEs in the extension phase (28%) was comparable with that in the double-blind phase (24.4%) and was the highest in the first 3 months of the extension phase. Constipation was assessed by the investigator as not being related to study medication in 2.9% of patients; therefore, the incidence of treatment-related constipation was 6.3%. Only 16% of patients used concomitant laxative medication during the extension phase and 8.7% had regular laxative intake. Diarrhoea was experienced by 3.2% of patients, although only 1.3% was considered to be related to oxycodone PR/naloxone PR.

**Table 4:** Incidence of adverse events reported by system organ class (≥ 10%) and preferred term (≥ 6%): analgesia study extension population

	Oxycodone PR/naloxone PR	
	*n* = 379	%
**Any adverse event**	258	68
GI disorders	106	28
Constipation	35	9.2
Nausea	29	7.7
**Infections and infestations**	82	22
Nasopharyngitis	20	5.3
**Musculoskeletal and connective tissue disorders**	79	21
Back pain	24	6.3
Nervous system disorders	53	14
**Psychiatric disorders**	41	11
Depression	24	6.3
Skin and subcutaneous tissue disorders	41	11
General disorders	39	10

The overall incidence of nervous system disorders was 14% (Table 4), being the highest in the first 3 months of the extension phase. Two patients reported an AE related to opioid withdrawal (drug withdrawal syndrome); however, both AEs started after the end of study medication intake and were, therefore, probably related to the change of opioid treatment. There were no treatment-related deaths during the course of the study.

Overall, 48 patients (13%) experienced 88 SAEs. Of these, 27 events in 12 patients were considered to have a causal relationship to study medication, but only six events in three patients were considered by the investigators to have a possible relationship to the study medication, and included confusional state, urge incontinence and depression in one patient, cholecystitis acute and cholelithiasis in one patient, and dyspepsia in one patient. No remedial action was deemed necessary in these three patients who recovered from these AEs. The sponsor also assessed the causality of the SAEs. In this regard, four SAEs were considered to have a stronger relationship to the study drug compared with the assessment of the investigator. A hypertensive crisis and epilepsy attack in two patients were assessed as possibly related, whereas arterial hypertension and abdominal pain in two other patients were assessed as unlikely related to the study drug. As a result, 30 events in 14 patients were considered by the sponsor to have a possible relationship with study medication. Overall, treatment was discontinued for four patients with SAEs that were considered causally related to the study drug.

After 6 and 12 months, the majority of the clinical laboratory values were normal. No clinically relevant changes in vital signs were observed during the study and ECG abnormalities were isolated.

### Bowel function study

A total of 258 patients (80.1%) of the 322 who were randomised in the double-blind phase entered the extension study and received the fixed combination oxycodone PR/naloxone PR. The majority of these patients (227, 88%) completed the study. Of the 31 patients (12%) who discontinued the study, 9 (3.5%) did so through choice, 4 (1.6%) did so because of administrative error and 3 (1.2%) were lost to follow up (Figure 2B). The cause of discontinuation was AEs in 12 patients (4.7%) and lack of therapeutic effect in three patients (1.2%).

Patients were 58.4 years in average, with 102 (39.5%) male and 156 (60.5%) female patients (Table 1). The average dose of oxycodone PR/naloxone PR received by the extension-phase population was 38.3 mg/day, which was not significantly higher than the average dose received in the double-blind study, in which the average dose of oxycodone PR was 34.0 mg/day and the average dose of oxycodone PR/naloxone PR was 32.8 mg/day.

#### Efficacy evaluation

**Figure 4:**
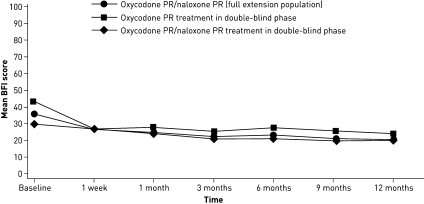
Mean BFI by visit: bowel function study for the LOCF full extension population (*n* = 258) and score according to double-blind phase treatment. BFI, Bowel Function Index; LOCF, last observation carried forward; PR, prolonged-release

The BFI score decreased throughout the extension phase. After 12 months of treatment with oxycodone PR/naloxone PR, in the LOCF analysis (*n* = 258), the mean BFI score (±SD) fell from 35.6 ± 27.74 at baseline (end of the double-blind study) to 20.6 ± 24.01 by 12 months, which represents an average 15-point reduction in BFI score (Figure 4). These results were supported by the non-LOCF analysis (*n* = 250), in which the mean BFI score (±SD) fell from 35.6 ± 27.74 at baseline to 20.4 ± 23.68 at 12 months.

A *post hoc* analysis was performed on the BFI results to categorise patients according to the treatment they received during the double-blind study. While all patients experienced improvements in BFI score throughout the extension phase, the greatest reduction in BFI was observed in those who switched from oxycodone PR to oxycodone PR/naloxone PR at the beginning of the extension phase. In these patients, the mean BFI score (±SD; based on the LOCF analysis) fell from 42.7 ± 28.61 at the start of the extension phase to 26.1 ± 23.31 after 1 week of treatment (Visit 10), compared with only a slight reduction from 28.7 ± 25.15 to 26.2 ± 25.09 respectively, for those who had received oxycodone PR/naloxone PR in the double-blind phase. From Week 1 onwards, the BFI fell at similar rates in the two groups (Figure 4). At the end of the extension phase, the mean BFI scores (±SD) decreased to 22.8 ± 25.59 and 18.6 ± 22.30 for patients who had previously received oxycodone PR only in the double-blind phase and those who had received oxycodone PR/naloxone PR respectively. The results were similar for the non-LOCF analysis.

After switching to the extension phase, the mean pain score (±SD) for the item ‘average pain over the last 24 h’ was 3.3 ± 1.77, which was similar to the combined mean pain score for the two treatment arms at the end of the double-blind phase (3.5 ± 1.87). The mean average pain scores (±SD) were similar at all visits in the extension phase, being 3.1 ± 1.94 at 12 months. During the first 7 days of treatment, 75.3% of patient days were free from use of analgesic rescue medication, and the mean (±SD) daily rescue dose was low (2.51 ± 4.60 mg). The mean (±SD) daily supplemental analgesic use was also low (0.4 ± 0.64) during Days 1–7 of the extension phase. For the remainder of the study, requirement for other opioid analgesic medication (at the investigator’s discretion) was reported by 83 patients (32.2%). During the first 7 days of the study, 24 patients (9.3%) received laxatives on a regular basis and after the first week, only 22 patients (8.5%) reported regular laxative intake.

#### Safety evaluation

A total of 211 patients (81.8%) experienced an AE during the extension phase (Table 5). Fewer than half of the patients (125; 48.4%) experienced AEs considered as having a causal relationship to study medication, which were classified as serious for only eight patients (3.1%). The most common AEs were infections and infestations (104 patients, 40.3%), and musculoskeletal and connective tissue disorders (104 patients, 40.3%). AEs led to study discontinuation in 3.5% of patients.

**Table 5:** Incidence of adverse events reported by system organ class (≥ 10%) and preferred term (≥ 6%): bowel function study extension population

	Oxycodone PR/Naloxone PR	
	(*n* = 258)	%
Any adverse event	211	81.8
**GI disorders**	94	36.4
Constipation	40	15.5
Diarrhoea	18	7.0
**General disorders and administration site conditions**	28	10.9
**Infections and infestations**	104	40.3
**Musculoskeletal and connective tissue disorders**	104	40.3
Arthralgia	23	8.9
Back pain	35	13.6
Osteoarthritis	16	6.2
**Nervous system disorders**	58	22.5
Headache	18	7.0
**Psychiatric disorders**	31	12.0
**Respiratory, thoracic and mediastinal disorders**	34	13.2
**Skin and subcutaneous tissue disorders**	41	15.9

Gastrointestinal AEs occurred in 94 patients (36.4%), with 40 (15.5%) experiencing constipation and 18 (7.0%) experiencing diarrhoea. There was one serious GI AE, although this case of abdominal pain was considered as unlikely to be related to treatment with oxycodone PR/naloxone PR. For patients who experienced constipation or diarrhoea, only 29 (11.2%) and 7 (2.7%), respectively, were classed as possibly, probably or definitely being related to treatment. Only three patients (1.2%) experienced an episode of severe diarrhoea. Treatment for constipation was given to 28 (10.9%) subjects but in no case did constipation result in treatment discontinuation, dose interruption or dose reduction.

One patient died during the study, but this was because of necrotising faciitis and was not related to treatment. A further 26 patients (10.1%) experienced 35 SAEs. Overall, 11 events in eight patients were considered by the investigators to have a possible relationship to the study medication and included cardiovascular disorder, ECG and angina pectoris in one patient, haematuria and bladder disorder in one patient, and atrial fibrillation, amnesia, non-cardiac chest pain, myocardial infarction, abdominal pain and cerebrovascular accident (one patient each). Only one SAE (amnesia) was considered possibly related to the study drug, for which treatment was discontinued. Treatment was also discontinued in the patient with the cerebrovascular accident. All eight patients recovered from the AE, although the patient with bladder disorder recovered with sequelae.

Subjective Opiate Withdrawal Scale score remained stable in the extension study, with the mean (SD) score being 4.7 (5.68) after 1 week of treatment and 6.0 (6.36) at the end of the study (12 months). End-of-study results for haematology, blood chemistry and vital sign parameters were statistically equivalent to their respective start-of-study values, as shown *post hoc* by means of two-sided TOST analyses on difference in [−1, +1] for basophils, eosinophils, total bilirubin and direct bilirubin, or ratio in [0.8, 1.25]. Abnormal blood pressure or liver enzyme levels were isolated. Changes in ECG were infrequent and isolated.

## Discussion

### Efficacy discussion

In the study focusing on analgesic efficacy, the mean score for the item ‘average pain over the last 24 h scores and BPI-SF pain subscores were low and stable over 6 and 12 months, indicating that oxycodone PR/naloxone PR provides effective long-term analgesic efficacy. These results were supported by the BPI-SF sleep quality item and interference subscores, which correlated with the low pain scores, indicating the positive effect of oxycodone PR/naloxone PR on sleep and activities – an effect that was maintained throughout the extension phase.

Following titration to an effective analgesic dose during the first 2 weeks of the extension study, the majority of patients remained on an oxycodone PR/naloxone PR dose comparable with that which they received in the double-blind study. There was no indication that the treatment that patients had received in the double-blind study influenced the dose increase after the first 2 weeks. Some patients did require an increase in dose; however, it is important to note that this increase in mean daily dose of oxycodone may reflect the natural progression of the underlying pain-causing condition, which is supported by the slight increase in dose observed during the 12-month extension phase. In addition, rescue medication intake was low, which could also represent an additional factor affecting the increase in study medication dose. A similar analgesic effect to that observed in the total study population was seen in the subpopulation of patients who received doses > 40/20 mg oxycodone PR/naloxone PR per day on > 7 days consecutively – mean pain scores were stable and comparable at all study visits throughout the extension phase. This also suggests that the long-term analgesic efficacy of oxycodone PR/naloxone PR was maintained at higher doses.

In this study focusing on bowel function as the main efficacy measure, the mean BFI scores continued to improve over 52 weeks of treatment with oxycodone PR/naloxone PR. Interestingly, patients who had previously received oxycodone PR in the double-blind phase experienced a rapid reduction in BFI score during the first week of treatment with the fixed combination of oxycodone PR/naloxone PR. Following this, BFI scores decreased at a similar rate to those in patients who had received oxycodone PR/naloxone PR in the double-blind study. The improvement in OIC was achieved without negatively affecting the analgesic efficacy of the oxycodone PR component, as mean pain scores remained low and stable, and use of rescue medication was low throughout the study. This supports the results from the extension study focusing on analgesic efficacy.

The results of these two extension studies show that the efficacy of the fixed combination of oxycodone PR/naloxone PR observed in the previous 12-week studies (22,23), in terms of both analgesia and improvements in bowel function, can be achieved and maintained during long-term therapy.

### Safety discussion

In the analgesia study, the incidence of AEs in the extension phase (68%) was comparable with that in the double-blind study (oxycodone PR/naloxone PR 55.8%, oxycodone PR 53.0%, placebo 52.5%) when the longer observation period was taken into account (22). The most frequently-reported AEs were either common side effects associated with opioid therapy [constipation (9.2%) and nausea (7.7%)] or potentially related to the underlying medical condition [back pain (6.3%) and depression (6.3%)]. Although 88 SAEs occurred in this study, only 27 events in 12 patients were considered to have a causal relationship with the study medication and only six events in three patients were considered possibly related, but no action was deemed necessary in terms of study medication changes. Indeed, all three patients recovered.

The incidence of GI AEs and nervous system disorders was the highest in the first 3 months of the analgesia study. In this sensitive phase, all subjects restarted opioid treatment with 20 mg oxycodone/naloxone, were uptitrated to their effective analgesic dose and started new analgesic co-medication (oxycodone IR as rescue medication). Throughout the next 9 months, the incidence of constipation consistently decreased, dropping from six subjects at 3 to 6 months to one subject at greater than 12 months. The incidence of constipation increased again to 12 subjects within 7 days after the end of oxycodone/naloxone treatment and the switch to a marketed product. In 11 subjects, constipation was assessed by the investigator as not related to study medication (2.9%). Therefore, the incidence of constipation related to study medication is reduced to 6.3%.

While 81.8% of patients experienced AEs in the bowel function study, only 48.4% of these events had a positive causal relationship to study medication; only 26 patients experienced SAEs, the majority of whom (*n* = 18) experienced SAEs that were not related to study medication. Of the eight SAEs considered to have a possible relationship with the study medication, only one was considered possibly related to the study medication (amnesia), and for which the treatment was discontinued and the patient recovered. Importantly, the incidence of diarrhoea was low, and SOWS sum scores were not exacerbated with oxycodone PR/naloxone PR.

Based on the AEs, clinical laboratory reports, and vital sign and ECG data, the long-term use of oxycodone PR/naloxone PR has a favourable tolerability profile. Furthermore, there was no indication of an increased risk of AEs in patients taking doses > 40/20 mg/day oxycodone PR/naloxone PR for > 7 days consecutively, compared with the total extension phase population.

### Discussion of study design, including choice of treatment groups and appropriateness of measurements

The study design allowed patients to be up-titrated to 80/40 mg oxycodone PR/naloxone PR per day and to take rescue medication. This ensured that patients received adequate pain relief similar to the usual practice of prescribing a dose of IR oxycodone, as needed, for pain. The efficacy measurements were those commonly used to evaluate pain in patients with chronic non-cancer pain and were consistent with other studies in this development programme. Moreover, the BFI (30,32) and the BPI-SF (29) with the interference subscore used as a QoL measure are validated instruments.

## Conclusion

Opioid-induced constipation is the most frequently reported AE experienced by patients receiving long-term opioid therapy, and can cause significant pain and discomfort. In many cases, this can be sufficiently severe to undermine the effectiveness of pain management and, therefore, negatively impact on patients’ QoL. The results from these studies provide evidence that the fixed combination of oxycodone PR/naloxone PR is a safe and efficacious agent for the long-term treatment of chronic pain. Mean scores for the item ‘average pain over the last 24 h’ and BPI-SF pain subscores remained low and stable throughout the 52-week study and were comparable with those observed at the end of the double-blind study (22), indicating good analgesic efficacy. In addition to delivering consistent analgesia throughout 52 weeks, oxycodone PR/naloxone PR continued to improve symptoms of OIC. This supports the long-term use of oxycodone PR/naloxone PR in the treatment of chronic pain. The AEs associated with oxycodone PR/naloxone PR are consistent with those generally observed with opioid therapy, and the combination of oxycodone PR with naloxone PR raises no additional safety concerns.
